# Multiclass Classification of Metrologically Resourceful Tripartite Quantum States with Deep Neural Networks

**DOI:** 10.3390/s22186767

**Published:** 2022-09-07

**Authors:** Syed Muhammad Abuzar Rizvi, Naema Asif, Muhammad Shohibul Ulum, Trung Q. Duong, Hyundong Shin

**Affiliations:** 1 Department of Electronics and Information Convergence Engineering, Kyung Hee University, Yongin 17104, Korea; 2 School of Electronics, Electrical Engineering and Computer Science, Queen’s University Belfast, Belfast BT7 1NN, UK

**Keywords:** quantum entanglement, quantum metrology, quantum sensing, Heisenberg limit, multiclass classification, artificial neural networks, deep neural networks

## Abstract

Quantum entanglement is a unique phenomenon of quantum mechanics, which has no classical counterpart and gives quantum systems their advantage in computing, communication, sensing, and metrology. In quantum sensing and metrology, utilizing an entangled probe state enhances the achievable precision more than its classical counterpart. Noise in the probe state preparation step can cause the system to output unentangled states, which might not be resourceful. Hence, an effective method for the detection and classification of tripartite entanglement is required at that step. However, current mathematical methods cannot robustly classify multiclass entanglement in tripartite quantum systems, especially in the case of mixed states. In this paper, we explore the utility of artificial neural networks for classifying the entanglement of tripartite quantum states into fully separable, biseparable, and fully entangled states. We employed Bell’s inequality for the dataset of tripartite quantum states and train the deep neural network for multiclass classification. This entanglement classification method is computationally efficient due to using a small number of measurements. At the same time, it also maintains generalization by covering a large Hilbert space of tripartite quantum states.

## 1. Introduction

Quantum information science (QIS) is an emerging area that includes storing, transmitting, processing, and measuring information using the principles of quantum mechanics. Under the domain of QIS, there are fundamental areas of science and engineering, which can be listed as quantum computation, quantum communication, quantum sensing, and quantum metrology [1,2,3]. In QIS, quantum sensing and metrology are relatively new fields. They focus on the use of quantum systems and quantum properties to measure a physical quantity. They can give greater accuracy and precision for measurements of physical quantities and detection of objects when compared to their classical analogs. Their applications can be found in communication technology, microscopy, electric and magnetic field sensing, gravity sensing, and many other areas [4].

The phenomena of quantum mechanics, such as superposition, interference, and entanglement are the foundation of QIS. In particular, quantum entanglement is a unique phenomenon with no classical analogy. It was a mysterious discovery that confused scientists for many years but also gave quantum systems the edge over classical systems [5]. A quantum system is considered entangled if the quantum states cannot be factored into the product of its local constituent states [6].

In quantum sensing and metrology, entanglement in the probe state can be very useful. This is prominent in the presence of noise where an entangled probe state can improve the precision scaling [7,8,9,10]. In particular cases, such as in the interferometric scene, tripartite entanglement is an essential resource that can surpass the classical limits of sensing and metrology, especially when entangled states are used, such as the Greenberger–Horne–Zeilinger (GHZ) state [7,11]. General steps involved in quantum sensing and metrology are: (i) state preparation, (ii) sensing, (iii) readout, and (iv) estimation. The task of state preparation can have imperfections and noise, causing the state produced to be separable or biseparable [12,13], which may not be resourceful for sensing and metrology tasks. Hence, the classification of quantum entanglement is critical at this step.

Conventional methods for detecting quantum entanglement involve quantum tomography to fully construct a density matrix, which requires a lot of measurements. Moreover, there is no universal method to detect all types of entanglement, especially in the case of tripartite mixed states, as it becomes difficult for simple mathematical formulations to model the large Hilbert space. Hence, alternate methods need to be explored.

Recently, researchers have attempted to combine the fields of quantum and machine learning; this new integrated area is termed ‘quantum machine learning’ [14]. The approaches to combining these areas can be divided into two different ways. One method is to utilize quantum algorithms to speed up classical machine learning [15]. While the other approach is to use classical machine learning to address the underlying challenges in QIS [16]. In this work, we explore the latter case, in which we use supervised machine learning to address the problem of quantum state classification. Artificial neural networks (ANNs) have appeared to simulate the dynamics of quantum systems effectively. Hence, they can be a powerful tool when dealing with quantum systems [17,18].

In this paper, we propose a solution for the entanglement classification of tripartite systems using an ANN. The method classifies pure and mixed tripartite states into three categories: fully separable, biseparable, and fully entangled. We train multiple ANNs, including a deep neural network (DNN), by extracting features of a density matrix based on Mermin and Svetlichny inequalities. Classification is done by finding an intricate relation between Bell’s inequality terms (as features) and the state’s label by using ANN. We show that by using the DNN, the entanglement of tripartite quantum states can be classified with good accuracy while using only the partial information of the quantum state. Hence, this is an effective method that can be employed in the probe state preparation step.

## 2. Preliminary

In this section, we give a brief overview of tripartite quantum entanglement, quantum sensing and metrology, ANNs, and Bell’s inequalities for feature extraction.

### 2.1. Quantum Entanglement

Entanglement is a phenomenon that was first discovered by Einstein and followed by Schrodinger; it marks the foundation of quantum physics. Entanglement occurs when two or more particles, demonstrating the laws of quantum physics, are allowed to interact in such a way that the quantum state of each individual particle or party involved in the entanglement process cannot be described independently of the state of others, even when the particles are very far away from each other. Quantum entanglement lies at the base of the distinction between classical and quantum physics and is a primary feature lacking in classical systems [19].

Entanglement has a variety of applications in QIS. With the help of entanglement, we can perform unique QIS tasks, such as quantum superdense coding [20] and quantum teleportation [21]. Quantum cryptography is another popular domain that utilizes entanglement to enable unconditionally secure communication [22]. In quantum computation, entanglement can provide polynomial or exponential speedups in some quantum algorithms and quantum simulations [23]. Quantum sensing and metrology use entangled particles to break past the standard quantum limit and take ultrasensitive measurements.

Bipartite entanglement is one of the basic forms of entanglement and is useful in understanding the entanglement concepts with quantum physics. Meanwhile, multipartite entanglement also has many applications and can have several advantages over bipartite entanglement. Some of its uses can be found in the formalization of quantum networks, execution of quantum computation in clusters, and also in the study of physics, such as many-body systems [24]. The simplest form of multipartite entanglement is the tripartite entanglement. Both the bipartite and tripartite entanglement will be discussed in this subsection.

#### 2.1.1. Bipartite Entanglement

A bipartite quantum state can either be separable or entangled. For ease of understanding and illustration, we will only discuss pure quantum states in this subsection. For demonstration, we will use the common notation of Alice and Bob, which will constitute the bipartite quantum system. The Hilbert space for this is denoted as $H_{A}^{2}⊗H_{B}^{2}$, where 2 shows that our system is two-dimensional.

(a) **Separable:** A bipartite pure state is called separable if it can be written as a single product of vectors that describe the subsystem states as:
  (1) $${|\psi^{s}\rangle }_{\mathrm{AB}}={|\psi \rangle }_{A}⊗{|\psi \rangle }_{B}.$$
(b) **Entangled:** A state that is not separable or that cannot be written as a product state is called entangled. For entangled states, we have complete knowledge about the whole system but our information about individual systems is incomplete. This information lies in the quantum correlations that were established due to the entanglement. One of the most fundamental and well-known types of bipartite entangled states are the Bell states, given as:
  (2) $${|\phi^{+}\rangle }_{\mathrm{AB}}=\frac{1}{\sqrt{2}}\left(|00\rangle +|11\rangle \right),$$
  (3) $${|\psi^{+}\rangle }_{\mathrm{AB}}=\frac{1}{\sqrt{2}}\left(|01\rangle +|10\rangle \right),$$
  (4) $${|\psi^{-}\rangle }_{\mathrm{AB}}=\frac{1}{\sqrt{2}}\left(|01\rangle -|10\rangle \right),$$
  (5) $${|\phi^{-}\rangle }_{\mathrm{AB}}=\frac{1}{\sqrt{2}}\left(|00\rangle -|11\rangle \right).$$
  The Bell states can be transformed into one another simply by employing a unitary transformation. Furthermore, these states are extensively used in QIS tasks, such as quantum teleportation, superdense coding, and secure quantum communication protocols.

#### 2.1.2. Tripartite Entanglement

**Figure 1:**
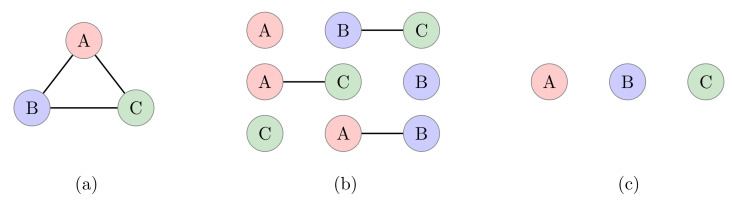
Tripartite entanglement classes depicting (**a**) fully entangled, (**b**) biseparable, and (**c**) fully separable systems.

There are three categories of tripartite quantum systems either pure or mixed, such as fully separable, biseparable, and fully entangled, as shown in Figure 1. The knowledge of mixed tripartite entanglement is much less advanced, so we will limit our discussion to pure states only. For demonstration, we will use the common notation of Alice, Bob, and Charlie, which will constitute the two-dimensional tripartite quantum system. The Hilbert space for this is denoted as $H_{A}^{2}⊗H_{B}^{2}⊗H_{C}^{2}$, where 2 shows that our system is two-dimensional.

(a) **Fully Separable:** In a fully separable case, there exists no entanglement between any of the parties involved in the composite systems. The tripartite pure state ${|\psi \rangle }_{\mathrm{ABC}}$ is fully separable if it can be written as a tensor product of each party’s state as:
  (6) $${|\psi^{\mathrm{fs}}\rangle }_{\mathrm{ABC}}={|\psi \rangle }_{A}⊗{|\psi \rangle }_{B}⊗{|\psi \rangle }_{C}.$$
(b) **Biseparable:** In this case, only two parties are entangled with each other in a bipartite fashion, as discussed in the previous subsection, while the remaining party is non-entangled. There are three possible cases for tripartite pure biseparable states, which can be written as:
  (7) $${|\psi^{\mathrm{bs}}\rangle }_{A|\mathrm{BC}}={|\psi \rangle }_{A}⊗{|\psi \rangle }_{\mathrm{BC}}$$
  where Bob and Charlie in ${|\psi \rangle }_{BC}$ are entangled. Similarly, the other cases are:
  (8) $${|\psi^{\mathrm{bs}}\rangle }_{B|\mathrm{AC}}={|\psi \rangle }_{B}⊗{|\psi \rangle }_{\mathrm{AC}},$$
  (9) $${|\psi^{\mathrm{bs}}\rangle }_{C|\mathrm{AB}}={|\psi \rangle }_{C}⊗{|\psi \rangle }_{\mathrm{AB}}.$$
(c) **Fully Entangled:** A tripartite state is called fully entangled if all three parties are entangled. In other words, the state is neither fully separable nor biseparable. The two states that represent the fundamental classes of maximally entangled tripartite states are the Greenberger–Horne–Zeilinger (GHZ) state and the W state, defined as:
  (10) $$|\mathrm{GHZ}\rangle =\frac{1}{\sqrt{2}}\left(|000\rangle +|111\rangle \right),$$
  (11) $$\;\;\;\;\;\;\;\;\;\;\;\;\;\;\;\;\;|W\rangle =\frac{1}{\sqrt{3}}\left(|100\rangle +|010\rangle +|001\rangle \right).$$
  These two types of states are totally inequivalent under stochastic local operations and classical communication. It means that it is impossible to transform any state of one class into any state of the other class. The entanglement of the GHZ state is very sensitive to noise errors. If any subsystem is traced out, the state will become a fully separable state. In contrast, the entanglement of the W state is more resilient, such that if any of the subsystems is traced out, the state will become a biseparable state.

### 2.2. Quantum Entanglement for Quantum Sensing and Metrology

Quantum sensing uses a quantum system to measure a physical quantity that can be classical or quantum. It exploits the main weaknesses of quantum systems, which are their sensitivities when exposed to the environment. Quantum sensing is a rapidly growing research field in QIS using common modalities, such as spin qubits, trapped ions, and flux qubits. Quantum sensing utilizes quantum properties with no classical analog, such as quantum entanglement, to surpass the precision scaling a classical sensor could achieve. General steps involved in quantum sensing and metrology are: (i) probe state preparation, (ii) sensing, (iii) readout, and (iv) estimation [4,13].

Entanglement-assisted sensing can elevate the precision scaling towards the fundamental limit governed by quantum theory, the Heisenberg limit. The study of using quantum resources to achieve this fundamental limit is called quantum metrology [4]. Hence, quantum sensing and metrology can be seen as the use of quantum systems to measure physical quantity utilizing quantum properties to achieve fundamental limits in precision. Entanglement-enhanced quantum sensing and metrology have found applications in a variety of fields. One example is the quantum frequency standards. It becomes prominent in spectroscopy, where measuring frequency or time with high accuracy is needed. This requires a very precise clock. The use of entangled cold ions can increase the precision of measured time by a factor of the squared root of the number of ions being used [25]. Another useful application is quantum lithography and microscopy [26,27], where using entanglement can be used to make photons with smaller wavelengths, enabling us to interact with and observe very small objects. Further, in quantum positioning and clock synchronization, entangling all the photons in the measurement beam can improve measurement accuracy [28].

The precision of quantum sensing and metrology can be seen in the quantum parameter estimation framework, where a parameter is encoded in a quantum system from its interaction with an external signal one wants to measure. Based on the measurement outcome, the estimation procedure is employed. However, the estimation will inherit statistical uncertainty due to the probabilistic nature of quantum systems. One can utilize an *n*-partite state to obtain the error with the scaling of $1/\sqrt{n}$, termed the standard quantum limit. The error scaling can be further reduced to 1/n, achieving the Heisenberg limit using quantum entanglement [29].

**Figure 2:**
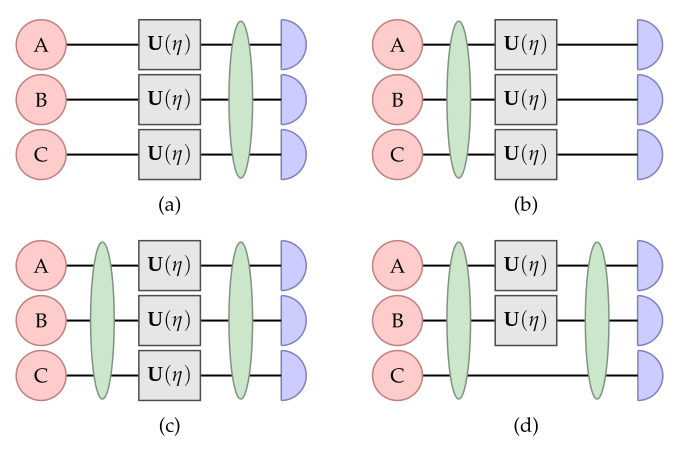
Different schemes depicting the use of quantum entanglement in quantum sensing and metrology experiments. Herein, the circle represents the probe state, the square represents the unitary evolution that encodes the unknown parameter η, the ellipse represents entangling operations, and the semicircle represents the measurement. (**a**) The quantum entanglement is only employed in the measurement, (**b**) the quantum entanglement is employed in the probe state, (**c**) both the probe state and measurement employ quantum entanglement, and (**d**) both the probe state and measurement employ quantum entanglement and the ancillary system is also included with entanglement.

There are several scenarios where we can use quantum entanglement in sensing and metrology tasks, as shown in Figure 2. That is, (i) quantum entanglement can be used in the probe state while the measurement remains classical; (ii) the probe state is classical, and the measurement has quantum entanglement; and (iii) both the probe state and measurement have quantum entanglement. It has been shown that the quantum entanglement in the probe state preparation is necessary to increase the precision scaling in sensing and metrology [30].

The encoding stage is done by a unitary evolution operator in the form of:

(12) $$\begin{array}{c} U\left(\eta \right)=e^{-\iota H\eta }, \end{array}$$

where $\iota =\sqrt{-1}$, H is the Hamiltonian of the system and η is the unknown parameter. The error of the estimator is bounded by [31,32]:

(13) $$\begin{array}{c} \Delta \eta \geq \frac{1}{2\Delta H}, \end{array}$$

where ${\left(\Delta H\right)}^{2}=\langle H^{2}\rangle -{\langle H\rangle }^{2}$ and 〈H〉=〈ψ|H|ψ〉. The minimum error of the estimator is achieved when ΔH is maximum, which is achieved for the state

(14) $$\begin{array}{c} |\psi \rangle =\frac{1}{\sqrt{2}}\left(|\lambda_{+}\rangle +|\lambda_{-}\rangle \right), \end{array}$$

where $\lambda_{+}$ and $\lambda_{-}$ are the maximum and minimum eigenvalues of H, respectively. For this probe state, ΔH has the maximum value of $\left(\lambda_{+}-\lambda_{-}\right)/2$.

To decrease the error, we use an *n*-partite probe state and evolve the state with $U{\left(\eta \right)}^{⊗n}$. Using the probe state ${|\psi \rangle }^{⊗n}$, the error will scale with $1/\sqrt{n}$ as $\Delta H=\sqrt{n}\left(\lambda_{+}-\lambda_{-}\right)/2$, the so-called standard quantum limit or classical limit. Using entanglement in the probe state can surpass this limit and achieve the Heisenberg limit. The entangled probe state that achieves the Heisenberg limit is given by:

(15) $$\begin{array}{c} |\psi^{\mathrm{en}}\rangle =\frac{1}{\sqrt{2}}\left(|\lambda_{+1}\rangle ⊗\cdots ⊗|\lambda_{+n}\rangle +|\lambda_{-1}\rangle ⊗\cdots ⊗|\lambda_{-n}\rangle \right), \end{array}$$

where $\lambda_{+\ell }$ and $\lambda_{-\ell }$ are the maximum and minimum eigenvalues of $H_{\ell }$ and ℓ∈{1,2,...,n} denotes the *ℓ*th party. The entangled probe state $|\psi^{\mathrm{en}}\rangle$ gives an error scaling of 1/n as $\Delta H=\sum_{\ell =1}^{n}\left(\lambda_{+\ell }-\lambda_{-\ell }\right)/2=n\left(\lambda_{+}-\lambda_{-}\right)/2$ and achieves the Heisenberg limit [33]. This probe state $|\psi^{\mathrm{en}}\rangle$ is a member of an equivalence class of the GHZ state, which is the optimal probe state in the noiseless system that can achieve the fundamental limit in sensing precision.

**Figure 3:**
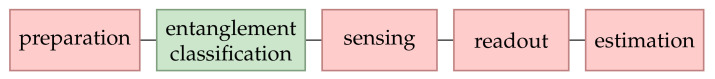
Stages in quantum sensing and metrology with an additional step for detection and classification of resourceful states.

Although the GHZ state can attain the precision limit in sensing and metrology, preparing such a state to realize the quantum advantage is hard due to imperfection in the preparation stage. For instance, the imperfect preparation of the GHZ state in trapped ${}^{9}{\mathrm{Be}}^{+}$ ions owing to the heating of the ions to a higher motional state will reduce its degree of entanglement [34], hence, limiting its quantum advantage. Moreover, the increasing decoherence rate also causes further problems. For example, in the uncorrelated dephasing noise, the decoherence rate of the GHZ state is *n*-times faster than the separable state. In depolarizing noise, the GHZ state entanglement can completely vanish, known as entanglement sudden death [12]. In general, imperfections and noise in the probe preparation stage can generate unentangled states. Hence, detecting and classifying the resourceful entanglement in the prepared probe state is important to harvest the quantum advantage in sensing and metrology. Therefore, quantum sensing and metrology stages must include a robust state classification step, as shown in Figure 3.

Furthermore, the fragility of the GHZ state motivates the search for other entangled states that are more robust against a noisy environment. In the presence of noise, partially entangled states may perform better than the GHZ state. Partially entangled states are more resilient to noise and have longer optimal sensing times. They also can be generated and measured more quickly as compared to GHZ states [35]. Therefore, having an entanglement classifier that can verify and classify such states can be useful.

#### 2.2.1. Tripartite Quantum Probe States

To witness the Heisenberg limit, one can use bipartite systems that are fully entangled. However, in specific scenarios, having just bipartite quantum systems is not enough to surpass the classical limit when there exist constraints in the experiment. For example, in interferometry, one needs at least three particles to beat the classical limit when one allows arbitrary power in the reference arm [7]. The study of tripartite entanglement becomes crucial in understanding the role of entanglement in quantum sensing and metrology. It is one step further than the simplest kind of entanglement, the bipartite entanglement, which only has two different classification types. In a tripartite state, the entangled systems can be further classified as biseparable and fully entangled. These two types can surpass the classical limit but have different effects on the precision scaling in quantum sensing and metrology, which we will discuss in the next subsection.

#### 2.2.2. Quantum Fisher Information

The quantitative measure of how resourceful a multipartite entanglement under the noise is well identified by quantum Fisher information, which has the following form:

(16) $$\begin{array}{c} F=2\sum_{i,j}\frac{{\left(\mu_{i}-\mu_{j}\right)}^{2}}{\mu_{i}+\mu_{j}}{\left|H_{ij}\right|}^{2}, \end{array}$$

where $\mu_{i}$ are the eigenvalues of the density matrix and $H_{ij}$ are the elements of the Hamiltonian matrix [36,37]. The quantum Fisher information serves as a lower bound for the variance of the estimator as ${\left(\Delta \eta \right)}^{2}\geq F^{-1}$. For pure states, the quantum Fisher information reads $F=4{\left(\Delta H\right)}^{2}$ whereas for mixed states, this inequality holds $F\leq 4{\left(\Delta H\right)}^{2}$. By taking $\lambda_{+}-\lambda_{-}=1$, the bound for tripartite fully separable states is [38]:

(17) $$\begin{array}{c} F\leq 3. \end{array}$$

Any state that violates this inequality contains entanglement. The quantum Fisher information for all tripartite states is bounded by:

(18) $$\begin{array}{c} F\leq 9. \end{array}$$

When the noise degrades the degree of entanglement, we can still bind the quantum Fisher information. For an *n*-partite probe state with *k* entangled parties at maximum, the quantum Fisher information is bounded by [8,39]:

(19) $$\begin{array}{c} F\leq mk^{2}+{\left(n-mk\right)}^{2}, \end{array}$$

where m=⌊n/k⌋. This bound is equal to 5 for the biseparable state in a tripartite quantum system. Hence, knowing whether the tripartite quantum state is fully separable, biseparable, or fully entangled will give us information about the achievable precision of the estimator.

### 2.3. ANNs and Supervised Learning

**Figure 4:**
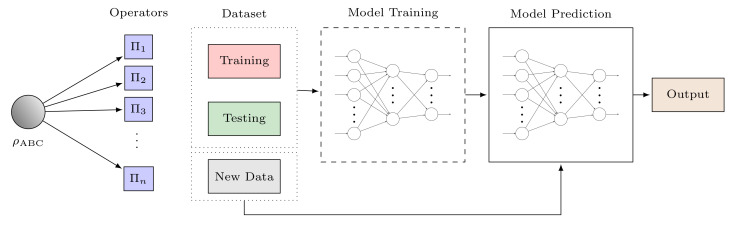
An ANN model for training, testing, and prediction.

The architecture of ANNs is similar to the biological structures of neurons in our brain; hence, the name ‘artificial neurons’. An ANN model consists of an input layer, hidden layers, and an output layer made of multiple neurons that are deeply connected. The neurons are based on activation functions, which fire the neuron based on the input threshold calculation in the previous layer. Training, testing, and validation data are used to train the model based on some optimizer. The general architecture of ANNs is shown in Figure 4.

In general, the ANN can be divided into three major learning paradigms, such as supervised learning, unsupervised learning, and reinforcement learning. In this work, we focus on supervised learning. Supervised machine learning refers to the set of learning methods in which both the data and the corresponding labels are provided as the input. In this scheme, we have a training set S containing paired values as follows:

(20) $$\begin{array}{c} S=\{\left(x_{1},y_{1}\right),\left(x_{2},y_{2}\right),\left(x_{3},y_{3}\right),\ldots \}, \end{array}$$

where $x_{i}$ and $y_{i}\in \left\{1,2,\ldots ,N\right\}$ denote feature vectors and the corresponding predetermined labels for *N* distinct classes, respectively. The objective of supervised learning for a pattern recognition task is to construct a classifier based on the training set that minimizes the cost function, which is the error between the network output and the desired output.

We apply the DNN-based supervised machine learning to solve the problem of quantum state classification. In general, it is a pattern recognition problem where we try to find the co-relations between a large dataset of randomly generated entangled and unentangled states lying between the discussed three categories of tripartite states. Using this mechanism, the DNN finds the hidden pattern in the data, which is intractable to find using other mathematical formulations. This is more important when dealing with quantum systems, where finding these correlations between states that are in superposition or are entangled is a challenging task.

The ANN models provide good descriptions of quantum systems [40,41]. This can be accredited to their power in reducing dimensionality and their ability to simulate higher-dimensional Hilbert spaces, which cannot be expressed or simulated by other analytical or numerical methods [42,43,44,45,46,47]. One such example mentions the use of ANNs in quantum tomography [17,48]. Herein, for tripartite quantum entanglement detection, we will input the data points in $x_{i}$ with the features that are extracted from the density matrices of quantum states, $\rho_{\mathrm{ABC}}$, acting on $H_{A}^{2}⊗H_{B}^{2}⊗H_{C}^{2}$. These features will be based on the inequality measures. Moreover, the quantum state classifier would output a label associated with the fully separable, biseparable, or fully entangled state. These labels are evaluated for the training set by employing various criteria, such as entanglement witness and partial positive transpose criteria.

### 2.4. Bell’s Inequality

In 1964, John Bell constructed an inequality that satisfies all the theories that are both local and counterfactual definite [5]. He proved that quantum mechanics behave differently and violates this inequality. This violation means that quantum mechanics cannot be both local and counterfactual definite. Therefore, based on the above assumptions, one can derive mathematical relations, i.e., Clauser–Horne–Shimony–Holt (CHSH) inequalities, which can be violated by the quantum mechanical systems [49]. Bell’s inequality can be constructed by considering a linear combination of products of observables, which is called the Bell operator. To reduce the number of terms in the inequality, we can use the expected value of this Bell operator rather than the joint probability of the outcomes. The following step is to calculate the maximum expected value that a system with local correlations can achieve and use it as the bound for the inequality. Any system violating this bound is then nonlocal and contains entanglement.

In this paper, we use each term in Bell’s inequality as a feature for the ANN to classify the entanglement of a tripartite quantum system. Specifically, we extract the features based on two widely-used Bell inequalities—namely, Mermin and Svetlichny inequalities. The ANN will generalize these inequalities in order to classify the entanglement of the quantum system.

#### 2.4.1. Bell’s Inequality for Bipartite Quantum Systems

Consider that Alice and Bob share a bipartite state $\rho_{\mathrm{AB}}$. Suppose that Alice has two observables $A_{0}$ and $A_{1}$, while Bob also has two observables $B_{0}$ and $B_{1}$ with possible outcomes as ±1 for each observable. We have the following CHSH inequality [49]:

(21) $$\begin{array}{c} \langle A_{0}B_{0}\rangle -\langle A_{0}B_{1}\rangle +\langle A_{1}B_{0}\rangle +\langle A_{1}B_{1}\rangle \leq 2, \end{array}$$

where 〈·〉 denotes the expectation. Quantum states violating the CHSH inequality can be labeled as ‘entangled’. The CHSH inequality is most violated by the maximally entangled state of the form

(22) $$|\psi^{\mathrm{en}}\rangle =\frac{1}{\sqrt{2}}\left(|00\rangle -|11\rangle \right),$$

which has the corresponding value of $2\sqrt{2}$. There exists no state that shows a higher violation, which is known as the Tsirelson bound [50]. We can consider a mixed state

(23) $$\begin{array}{c} \rho^{\mathrm{en}}=p|\psi^{\mathrm{en}}\rangle \langle \psi^{\mathrm{en}}|+\frac{1-p}{4}I, \end{array}$$

which is still entangled for p≥1/3 but the CHSH inequality is violated only for $p\geq 1/\sqrt{2}$, where I is the identity matrix. Hence, there exist entangled states that do not violate the CHSH inequality. However, it can detect all entangled pure states. Another drawback of this inequality is that the measurement angles depend on the quantum state.

To detect more states, we can introduce more two-outcome measurement settings [51]. For example, each party can measure three two-outcome measurements (i.e., $A_{i}$ for Alice and $B_{i}$ for Bob, i=0,1,2) and use the following inequality:

(24) $$\begin{array}{cc} \langle A_{0}I\rangle -\langle A_{1}I\rangle & +\langle IB_{0}\rangle -\langle IB_{1}\rangle +\langle A_{0}B_{0}\rangle -\langle A_{0}B_{1}\rangle +\langle A_{1}B_{0}\rangle \\ & +\langle A_{1}B_{1}\rangle +\langle A_{0}B_{2}\rangle +\langle A_{1}B_{2}\rangle +\langle A_{2}B_{0}\rangle +\langle A_{2}B_{1}\rangle \leq 4. \end{array}$$

However, it increases the number of measurements.

#### 2.4.2. Mermin Inequality

Bell’s inequality for bipartite systems can be generalized to multipartite systems. Similar to the CHSH inequality, which uses two measurement settings for each party in bipartite systems, there is the Mermin inequality for multipartite systems [52]. For three-qubit systems, the Mermin inequality has the form:

(25) $$\begin{array}{c} \langle A_{0}B_{0}C_{0}\rangle -\langle A_{0}B_{1}C_{1}\rangle -\langle A_{1}B_{0}C_{1}\rangle -\langle A_{1}B_{1}C_{0}\rangle \leq 2. \end{array}$$

The Mermin inequality is most violated by the GHZ state. For a noisy GHZ state in the form of:

(26) $$\rho^{\mathrm{GHZ}}=p|\mathrm{GHZ}\rangle \langle \mathrm{GHZ}|+\frac{1-p}{8}I,$$

the Mermin inequality can detect the entanglement when p≥0.5 [53]. Similar to the CHSH inequality, it cannot detect all of the entangled states. The detection may be increased by adding the number of measurement settings.

#### 2.4.3. Svetlichny Inequality

The Svetlichny inequality—a double tripartite Mermin inequality or a double CHSH inequality when two parties are combined—is given as [54]:

(27) $$\begin{array}{cc} -\langle A_{0}B_{0}C_{0}\rangle & +\langle A_{0}B_{0}C_{1}\rangle +\langle A_{0}B_{1}C_{0}\rangle +\langle A_{0}B_{1}C_{1}\rangle \\ \; & +\langle A_{1}B_{0}C_{0}\rangle +\langle A_{1}B_{0}C_{1}\rangle +\langle A_{1}B_{1}C_{0}\rangle -\langle A_{1}B_{1}C_{1}\rangle \leq 4. \end{array}$$

This inequality is developed to detect genuine tripartite non-locality. The violation of this inequality is a sufficient but not necessary condition to detect genuine tripartite nonlocal correlations. However, it cannot detect all genuine tripartite entanglements even for pure states.

## 3. Methods

We focus on classifying tripartite quantum states into fully separable, biseparable, and fully entangled states by constructing a classifier based on inequalities. This helps us to build a classifier using partial information instead of full information of quantum states. Our main strategy is to use the Mermin and Svetlichny inequalities to extract partial information about the quantum state. We use this information to train an ANN that can correctly classify an unknown quantum state into its respective class.

### 3.1. Optimizing Mermin and Svetlichny Inequalities

We consider the following measurement settings based on Pauli operators for the three parties (namely, Alice, Bob, and Charlie):

(28) $$\begin{array}{cc} \; & \left\{\begin{array}{c} A_{0}=\sigma_{x}\\ B_{0}=-\sigma_{y}\\ C_{0}=\sigma_{x}, \end{array}\right. \end{array}$$

(29) $$\begin{array}{cc} \; & \left\{\begin{array}{c} A_{1}=\sigma_{z}\\ B_{1}=\sigma_{z}\\ C_{1}=\sigma_{z}, \end{array}\right. \end{array}$$

where $\sigma_{x}$, $\sigma_{y}$, and $\sigma_{z}$ are Pauli *x*, *y*, and *z* operators, respectively. For the operators in (28) and (29) to work as classifiers, we introduce a weight on each term of the Mermin operator as follows:

(30) $$\begin{array}{c} \Pi_{\mathrm{Mermin}-\mathrm{ML}}=w_{0}A_{0}B_{0}C_{0}+w_{1}A_{0}B_{1}C_{1}+w_{2}A_{1}B_{0}C_{1}+w_{3}A_{1}B_{1}C_{0}, \end{array}$$

where the weights $w_{0}$, $w_{1}$, $w_{2}$, and $w_{3}$ are obtained by training the neural network. Hence, for a given tripartite quantum state, the set:

$$\left\{\langle A_{0}B_{0}C_{0}\rangle ,\langle A_{0}B_{1}C_{1}\rangle ,\langle A_{1}B_{0}C_{1}\rangle ,\langle A_{1}B_{1}C_{0}\rangle \right\}$$

is taken as the Mermin feature set of the ANN, having four operators. Each operator acts on a tripartite quantum state to give a unique feature.

Similarly, by introducing weights on the individual terms of the Svetlichny operator, we have:

(31) $$\begin{array}{cc} \Pi_{\mathrm{Svetlichny}-\mathrm{ML}} & =v_{0}A_{0}B_{0}C_{0}+v_{1}A_{0}B_{0}C_{1}+v_{2}A_{0}B_{1}C_{0}+v_{3}A_{0}B_{1}C_{1}\\ \; & \;+v_{4}A_{1}B_{0}C_{0}+v_{5}A_{1}B_{0}C_{1}+v_{6}A_{1}B_{1}C_{0}+v_{7}A_{1}B_{1}C_{1}, \end{array}$$

where the weights $v_{i}$, i=0,1,…,7, are obtained again by training the neural network. Here, we can take the set:

$$\left\{\langle A_{0}B_{0}C_{0}\rangle ,\langle A_{0}B_{0}C_{1}\rangle ,\langle A_{0}B_{1}C_{0}\rangle ,\langle A_{0}B_{1}C_{1}\rangle ,\langle A_{1}B_{0}C_{0}\rangle ,\langle A_{1}B_{0}C_{1}\rangle ,\langle A_{1}B_{1}C_{0}\rangle ,\langle A_{1}B_{1}C_{1}\rangle \right\}$$

as the Svetlichny feature set of the ANN, having eight operators. Each operator contributes to eight different features of a tripartite quantum state.

Therefore, we can consider a class of classifiers for tripartite quantum states, denoted as $\mathrm{Bell}-\mathrm{DNN}\left(n_{f},n_{d},n_{h}\right)$, where $n_{f}$, $n_{d}$, and $n_{h}$ denote the number of features, the number of hidden layers, and the number of neurons in each hidden layer, respectively. For Mermin features, $n_{f}=4$; for Svetlichny features, $n_{f}=8$. The integration of inequalities with machine learning for tripartite quantum state classification is summarized in Table 1.

### 3.2. Generating Quantum Datasets and Labels

To obtain the feature vector for the training of our ANN, we generate mixed tripartite quantum states for the three classes. The data generation and feature extraction for all cases were executed using the MATLAB toolbox for exploring quantum entanglement theory—QETLAB [55,56].

The function ‘RandomDensityMatrix(*d*)’ from QETLAB generates a random d×d density matrix, distributed according to the Hilbert–Schmidt measure. The function is designed to generate entangled quantum states.

If the given dimension to the function is 2, then it will output a simple random density matrix for a single qubit. If the dimension is changed to 4, it will output an entangled bipartite random density matrix. If the dimension is changed to 8, it will output a fully entangled tripartite random density matrix. We could further control how these density matrices would look, for example, mixed or pure, by changing the parameters of the function.

For fully separable quantum states labeled as 1, we generate a dataset of $10^{6}$ samples. Three single-qubit 2×2 dimension mixed quantum states are randomly generated by the ‘RandomDensityMatrix’ and then these states are tensored to give a fully separable tripartite quantum state as given by (6). For biseparable quantum states labeled as 2, we generate an entangled bipartite quantum state of dimension 4×4 using the RandomDensityMatrix and tensor it with a random single-qubit 2×2 state as given by (7)–(9), to construct a dataset of $10^{6}$ samples. Finally, for entangled tripartite quantum states labeled as 3, we construct a dataset of $10^{6}$ by using RandomDensityMatrix to output a random tripartite state of dimension 8×8, which will be fully entangled by default as the function always gives entangled bipartite and tripartite entangled states. The overall dataset is of size $3\times 10^{6}$. For comparison, we also generate datasets for the classification of pure tripartite quantum states using the same method and changing the parameters of the ‘RandomDensityMatrix’ function. The Mermin and Svetlichny features were also calculated in MATLAB by taking the projection of individual operator terms of inequalities and the density matrices.

### 3.3. Training the ANN

**Figure 5:**
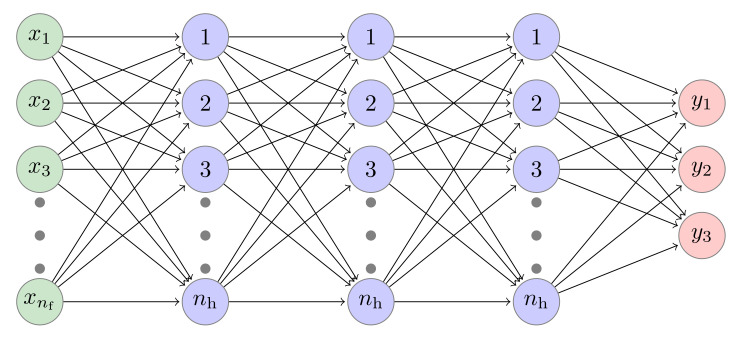
A DNN model with three hidden layers ($n_{d}=3$) for classification of tripartite states. Green nodes represent input units where the number $n_{f}$ of features is equal to 4 for Mermin and 8 for Svetlichny inequality. Blue nodes represent the hidden nodes where the ReLu is chosen as the activation function. Red nodes represent the output units where Softmax is used as the activation function.

We utilize the ANN as our machine learning method to design the classifier. The input units of our model are 4 and 8 neurons for features extracted from Mermin and Svetlichny inequalities, respectively. The generated dataset is divided into training and testing sets in the ratio 8:2. The training data were further divided into the training and validation data with the ratio of 8:2. For comparison purposes, we first construct and train the simplest neural network consisting of a linear connection and nonlinear output with the Softmax function that converts a vector of numbers into a vector of probabilities at its output layer. the Softmax function is defined as:

(32) $$\sigma {\left(z\right)}_{i}=\frac{e^{z_{i}}}{\sum_{j=1}^{K}e^{z_{j}}},$$

where σ is Softmax, $z=\left[z_{1},z_{2},\ldots ,z_{K}\right]$ is the input vector, $e^{z_{i}}$ is the standard exponential function for the input vector, *K* is the number of classes, and $\sum_{j=1}^{K}e^{z_{j}}$ is the normalization term to ensure that output values of the function sum to one. Afterward, we introduce nonlinearity to the model by adding a dense hidden layer with a different number of neurons and ‘rectified linear unit’ (ReLu) as its activation function. ReLu is a piecewise linear function that will output the input directly if it is positive otherwise it will output zero, which is defined as:

(33) f(x)=max(0,x),

where *x* is the input to the function. To further improve the classification accuracy, we introduce several dense hidden layers (see Figure 5) and observe the effect on the performance. The model was trained for 100 passes of the entire training dataset or epochs to obtain the weight parameters of the classifier. We used the loss function, which is a method of evaluating how the algorithm models the dataset, to be categorical cross-entropy, given as:

(34) $$L_{\mathrm{CE}}=-\sum_{i=1}^{K}t_{i}\log\left(p_{i}\right),$$

where $t_{i}$ is the truth label, $p_{i}$ is the Softmax probability for the $i^{th}$ class, and *K* is the number of classes. The optimizer for training was taken to be ‘root mean square propagation’ (RMSprop) to increase our learning rate for faster convergence. Callbacks with early stopping were also implemented to monitor when the model stopped improving to obtain the best one.

## 4. Results

In this section, we provide numerical results and discuss the implications of our proposed method to address the problem of quantum state classification for tripartite quantum states. To evaluate the performance of the machine learning model, we utilized different metrics. The common method is to find the ‘accuracy’ of the model. This is an intuitive performance measure, and it is simply a ratio of correctly predicted observations to the total observations, given as:

(35) $$\mathrm{Accuracy}=\frac{\mathrm{TP}+\mathrm{TN}}{\mathrm{TP}+\mathrm{TN}+\mathrm{FP}+\mathrm{FN}},$$

where TP (true positive) is the positive sample that is correctly predicted, TN (true negative) is the negative sample that is correctly predicted, FP (false positive) is the positive sample that is predicted incorrectly, and FN (false negative) is the negative sample that is predicted incorrectly. We will also see the ‘precision’ of the models, which measures the proportion of positively predicted labels that are actually correct, given as:

(36) $$\mathrm{Precision}=\frac{\mathrm{TP}}{\mathrm{TP}+\mathrm{FP}}.$$

Similarly, we also used ‘recall’, which is the ratio of correctly predicted positive observations to all the observations in the actual class, given as:

(37) $$\mathrm{Recall}=\frac{\mathrm{TP}}{\mathrm{TP}+\mathrm{FN}}.$$

Finally, we calculate the ‘F1-Score’, which represents the model score as a function of the precision and recall score, given as:

(38) $$F1-\mathrm{Score}\;=\frac{2\times \mathrm{Precision}\times \mathrm{Recall}}{\mathrm{Precision}+\mathrm{Recall}}.$$

To calculate the average performance, we used macro average, which is a simple average of the performance scores of each class. We will also see each model’s loss value, which shows how good or bad a model behaves after each iteration of optimization.

### 4.1. Linear Optimization

Using the ANN for linear optimization gives us unsatisfactory results. The classifier model $\mathrm{Bell}-\mathrm{DNN}\left(4,0,-\right)$ for Mermin features gives a very low accuracy of 32.10% and a high loss value of 1.0986. Similarly, for Svetlichny features, the $\mathrm{Bell}-\mathrm{DNN}\left(8,0,-\right)$ produces an accuracy of 37.19% and a loss value of 1.0987. Therefore, the linear optimization of the inequalities cannot provide the required state classification solution for tripartite quantum states.

### 4.2. Nonlinear Optimization

**Figure 6:**
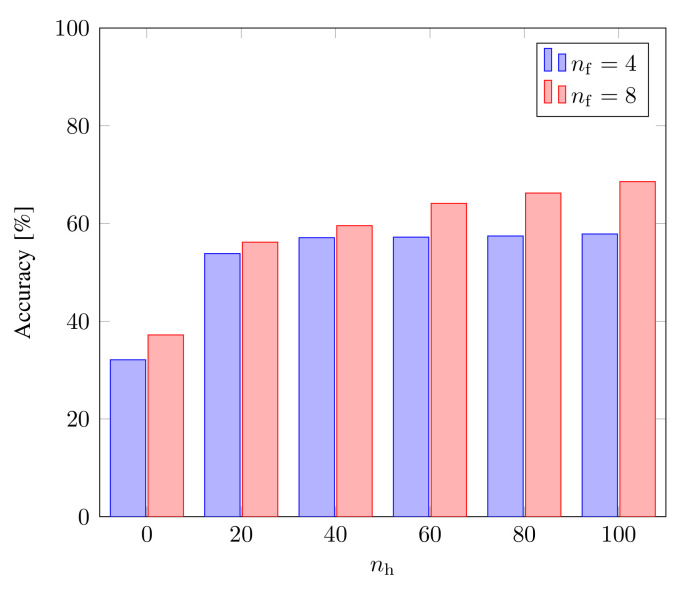
Classification accuracy as a function of the number of hidden neurons, $n_{h}$ in a single hidden layer ($n_{d}=1$) for Mermin ($n_{f}=4$) and Svetlichny ($n_{f}=8$) features. No hidden neuron ($n_{h}=0$) corresponds to linear optimization with no hidden layer.

To design a better classifier, we trained a neural network with a hidden layer for both inequalities. We chose the number of hidden neurons as $n_{h}=20,40,60,80,100$. We observe that the hidden layer improves the accuracy to 57.85% for $\mathrm{Bell}-\mathrm{DNN}\left(4,1,100\right)$ and 68.57% for $\mathrm{Bell}-\mathrm{DNN}\left(8,1,100\right)$, as shown in Figure 6. However, a further increase in the hidden neurons shows no significant improvement in the classification accuracy. The classifiers designed with 8 features from the Svetlichny inequality outperform the classifiers built with 4 features from the Mermin inequality due to the increase in relevant features, as expected.

### 4.3. Deep Learning Classifiers

To improve the classification of tripartite quantum states, we built and trained classifiers using a DNN with three dense hidden layers as shown in Figure 5 and gained an increase in the performance for Svetlichny features. For Mermin features, the deep learning techniques could not further improve the classification performance due to the fact that only four features were involved in training the neural network. Hence, we focused on the results of the model trained by Svetlichny features.

**Figure 7:**
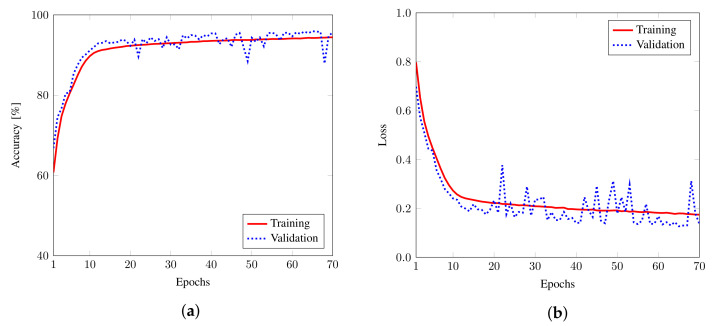
Training/validation (**a**) accuracy and (**b**) loss of the $\mathrm{Bell}-\mathrm{DNN}\;\left(8,3,80\right)$ classifier as a function of the number of epochs for Svetlichny features.

**Figure 8:**
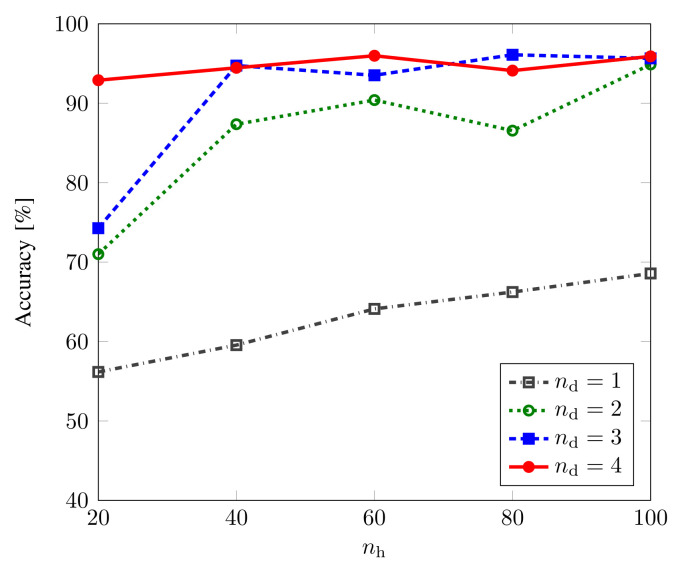
Classification accuracy of the $\mathrm{Bell}-\mathrm{DNN}\;\left(8,n_{d},n_{h}\right)$ model as a function of the number of hidden neurons, $n_{h}$ for Svetlichny features when $n_{d}=1,2,3,4$.

Figure 7 shows the training/validation accuracy and loss with respect to epochs for the $\mathrm{Bell}-\mathrm{DNN}\;\left(8,3,80\right)$ classifier with Svetlichny features. We observe that, as the model was trained, the accuracy increased to 96.10% with the loss decreasing to 0.1281 for Svetlichny features. Figure 8 shows the classification accuracy of the $\mathrm{Bell}-\mathrm{DNN}\;\left(8,n_{d},n_{h}\right)$ model as a function of $n_{h}$ when $n_{d}=1,2,3,4$. It was observed that 3 hidden layers with 80 neurons in each layer were sufficient for tripartite classification with good accuracy using 8 Svetlichny features.

The deep learning performance of these classifiers can be further ascertained by referring to Table 2 and Table 3 where the classification reports are given for the $\mathrm{Bell}-\mathrm{DNN}\;\left(8,3,80\right)$ models trained with Svetlichny features for mixed and pure tripartite quantum states, respectively. From the F1-scores, we observe that classifying biseparable states is more difficult than the classification of fully separable or fully entangled states.

## 5. Discussion and Conclusions

Quantum sensing and metrology can provide ultra-sensitive and precise measurements. To achieve this, entanglement is a key resource in the probe state preparation. Near-term quantum sensors will depend on the ability to generate and classify a variety of multipartite entangled states [57]. Hence, finding an entanglement classifier that can classify multipartite entanglement is crucial for establishing future quantum sensing devices. For the case of tripartite quantum states, there is no universal classification method of entanglement. The most popular mathematical method for classification, the positive partial transposition (PPT) criterion (or Peres–Horodecki criterion), applies only to bipartite systems. Other analytical methods are either unreliable or lack universality. For instance, a nonlinear entanglement witness can be used for entanglement classification but it can only detect a fraction of mixed states [58]. Likewise, methods such as concurrence calculation can also be used to classify the tripartite states, but they require complete quantum state tomography to reconstruct the density matrix, which scales exponentially with the number of parties constituting the overall quantum system [59]. For an *n*-qubit system, the number of measurements required to universally classify the entanglement is equal to $4^{n}-1$, making it 63 for the tripartite quantum systems. Similarly, Mermin and Svetlichny inequalities can be used for the said purpose. However, these can only detect some specific types of entanglement. There has been some work done to classify entanglement using neural network quantum states [60,61]. However, these methods rely on the ability of the network to construct the desired states efficiently and fall behind where the quantum system is too complex.

In this work, we constructed a simple, universal, and robust entanglement classifier for tripartite states using ANN. Using Bell’s inequality as a feature, we can reduce the number of measurements for classifying tripartite states. Specifically, for Mermin and Svetlichny inequalities, the measurement numbers were reduced to 4 and 8, respectively. We showed that DNN could classify tripartite quantum states with great accuracy, robustness, and universality while using only eight measurements. Using the inequalities, ANN acted as a classifier containing many entanglement witnesses. An entangled quantum state may not violate some entanglement witness to be classified as entangled, but it will definitely violate at least one of the entanglement witnesses encoded into the ANN to be classified correctly. Therefore the higher the number of entanglement witnesses encoded into the network, the higher our model’s accuracy will be. Hence, using classifiers with 8 features from the Svetlichny inequality outperformed the 4 features from the Mermin inequality. Furthermore, for the pure states, which have a less complex Hilbert space, our DNN models can classify them with greater accuracy, as shown in Table 3. These models can be scaled up for detecting high-dimensional quantum entanglement if we can find a reliable and efficient labeling criterion for the desired entanglement classes in high-dimensional multipartite quantum systems. 

## Figures and Tables

**Table 1:** Integrating Mermin and Svetlichny inequalities with machine learning.

Operator	Feature	Weight	Architecture
Mermin	fixed	4 fixed values	linear, no hidden layer
Svetlichny	fixed	8 fixed values	linear, no hidden layer
Mermin-ML	variable	many optimized values	nonlinear, hidden layer
Svetlichny-ML	variable	many optimized values	nonlinear, hidden layer

**Table 2:** Classification report for the $\mathrm{Bell}-\mathrm{DNN}\;\left(8,3,80\right)$ model trained with Svetlichny features for mixed tripartite quantum states.

Class	Precision	Recall	F1-Score
Fully Separable	0.92	1	0.96
Biseparable	0.96	0.92	0.94
Fully Entanglement	1	0.96	0.98
Accuracy	N/A	N/A	0.96
Macro Avg	0.96	0.96	0.96

**Table 3:** Classification report for the $\mathrm{Bell}-\mathrm{DNN}\;\left(8,3,80\right)$ model trained with Svetlichny features for pure tripartite quantum states.

Class	Precision	Recall	F1-Score
Fully Separable	0.95	1	0.97
Biseparable	0.96	0.94	0.95
Fully Entanglement	0.99	0.97	0.98
Accuracy	N/A	N/A	0.97
Macro Avg	0.97	0.97	0.97

## Data Availability

The data generated from the empirical results and the source code that support the findings of this study are available from the corresponding author upon reasonable request.

## Abbreviations

The following abbreviations are used in this manuscript:

QIS	quantum information science
ANN	artificial neural network
DNN	deep neural network
GHZ	Greenberger–Horne–Zeilinger
ReLu	rectified linear unit
RMSprop	root mean square propagation
PPT	positive partial transpose
CHSH	Clauser–Horne–Shimony–Holt
